# An Optimized Probabilistic Delay Tolerant Network (DTN) Routing Protocol Based on Scheduling Mechanism for Internet of Things (IoT) †

**DOI:** 10.3390/s19020243

**Published:** 2019-01-10

**Authors:** Yuxin Mao, Chenqian Zhou, Yun Ling, Jaime Lloret

**Affiliations:** 1 School of Management and E-Business, Zhejiang Gongshang University, Hangzhou 310018, China; susiezcqzj@126.com; 2 School of Computer and Information Engineering, Zhejiang Gongshang University, Hangzhou 310018, China; yling@mail.zjgsu.edu.cn; 3 Department of Communications, Universidad Politecnica de Valencia, 46730 Valencia, Spain; jlloret@dcom.upv.es

**Keywords:** PROPHET routing, delay tolerant network, scheduling mechanism, ONE simulator, Internet of Things

## Abstract

Many applications of Internet of Things (IoT) have been implemented based on unreliable wireless or mobile networks like the delay tolerant network (DTN). Therefore, it is an important issue for IoT applications to achieve efficient data transmission in DTN. In order to improve delivery rate and optimize delivery delay with low overhead in DTN for IoT applications, we propose a new routing protocol, called Scheduling-Probabilistic Routing Protocol using History of Encounters and Transitivity (PROPHET). In this protocol, we calculate the delivery predictability according to the encountering frequency among nodes. Two scheduling mechanisms are proposed to extend the traditional PROPHET protocol and improve performance in both storage and transmission in DTN. In order to evaluate the proposed routing protocol, we perform simulations and compare it with other routing protocols in an Opportunistic Network Environment (ONE) simulator. The results demonstrate that the proposed Scheduling-PROPHET can achieve better performances in several key aspects compared with the existing protocols.

## 1. Introduction

Traditionally, the internet is formed primarily by connectivity homogeneous devices (i.e., computers). But, nowadays, there are several paradigms in networking such as mobile, grid and cloud computing, which enable a purposeful connectivity between various devices such as computers, smart-phones, tablets, cameras, etc. As a new paradigm for this vision, the Internet of Things (IoT) is introduced in the area of wireless communications and networking [1]. IoT refers to the emerging trend of expanding hand-held devices and physical objects with sensing, computing and communication capabilities, connecting them to form a network. The IoT research covers a wide range of areas, including object identification and tracking, object networking, sensing data visualization, privacy control, and so on. With the advent of IoT, the underlying networks play a more significant role for data transmission in IoT applications. However, many IoT applications just suffer from problems such as unreliable wireless, poor trust model and weak mobile networks. An IoT system in an urban environment is often characterized by a lack of connectivity, dynamic mobility and long links interruptions. These unstructured networks can be considered as a type of delay/disruption tolerant networks (DTN) [2,3]. In some specific circumstances like InterPlaNetary internets, military ad-hoc networks, pocket switched networks, vehicular ad-hoc networks, mobile social networks and so forth, DTN has shown its high reliability since it owns the ability of data storage and transmission at each node.

In DTN, the end-to-end connectivity between source nodes and destination nodes rarely exists. Due to this disruption connectivity, messages should be forwarded to intermediate participating nodes by using the “store-carry-forward” mechanism, if the source node intends to deliver messages to the destination node. Moreover, time is limited for intermediate participating nodes to transfer messages. That is why traditional routing protocols cannot work well in the “store-carry-forward” mechanism. Therefore, it raises a serious challenge in designing efficient routing protocols for DTN in many IoT applications. An efficient routing protocol in DTN has a direct contextual relevance to the data transmission in many IoT applications. In recent years, numerous protocols were proposed to maximize delivery success, such as First Contact [4], Epidemic [5], Spray and Wait [6], Probabilistic Routing Protocol using History of Encounters and Transitivity (PROPHET), [7] etc. Among them, PROPHET is a typical routing protocol based on historical data. However, there are some drawbacks in PROPHET [7]:

- The delivered messages may be damaged by malicious intermediate nodes on the way from source to destination.
- Messages are more likely to be aborted as there are too many intermediate nodes and hops on connected paths.
- Due to TTL (Time to Live) and buffer congestion, some messages may be dropped on the way.

In classic PROPHET, when two nodes encounter frequently, the delivery predictability will increase rapidly. However, if two nodes have no chance to encounter due to network failures in some time slots, the delivery predictability will decrease quickly. Furthermore, messages may be broken, dropped or aborted, due to the drawbacks of the management and transmission. In order to address the issues above and improve the PROPHET protocol, we propose a new routing protocol called Scheduling-PROPHET in this study, which can support more efficient routing performance in DTN for IoT applications. The main superiority of Scheduling-PROPHET is to improve the efficiency of transmission with low message lost and high delivery rate. The remainder of the study is organized as follows. Section 2 gives an overview of related work and presents the motivation for doing the research. In Section 3, we propose a new protocol in order to improve the PROPHET routing and illustrate our methods in detail. Section 4 presents the simulation results as well as the discussion. Finally, we conclude the paper briefly in Section 5.

## 2. Related Work

In DTN, routing protocols aim to achieve a high message delivery rate and optimal delay, while maintaining low overhead. There have been numerous routing protocols proposed for DTN, with their own characteristics and optimal goals, and they are used in corresponding scenarios. Epidemic [5] is a traditional routing protocol based on flooding messages throughout the network. Its basic principle is to exchange messages with all nodes, so called epidemic, but it always causes a waste of resources. A more efficient way to perform routing in DTN is PROPHET [7]. Lindgren et al. designed a probabilistic routing protocol, which select intermediate nodes based on historical encounters and transmission.

The challenge of a routing protocol is always an active research area in DTN. Recently, there are two key factors considered in designing routing protocols, which are encountering opportunity and node mobility. Some researchers studied on the node movement environment. They described that the area of the nodes’ movement and encountering opportunity are strongly related to their locations at a particular time, and improve performance [8,9]. In a similar way, Refs. [10,11] select nodes with physical location and mobility behavior. Some routing protocols calculate transmission reliability by using history information such as encounters [7,12,13]. Bulut et al. calculates the average encountering time to select the nodes [14]. Social-based approaches attempt to exploit the social behaviors of DTN nodes to make better routing decisions. Li et al. in Ref. [15] propose a social-energy-based routing protocol that considers the social energy of encountering nodes and is in favor of the node with a higher social energy in its or the destination’s social community. Basilico et al. in Ref. [16] focus on the DTN routing without a priori mobility knowledge and provide approximation algorithms with theoretical guarantees that can be applied to cases where the number of hops allowed in the routing process is arbitrary. Some other routing protocols are about simply-copy in transmission [17,18]. The GTDM (Game Theory Based Decision Making) routing protocol improves performance based on game theory [19]. Sakai et al. in Ref. [20] design an abstract of anonymous routing protocols for DTNs and augment the existing solution with multi-copy message forwarding. They construct simplified mathematical models, which can be used to understand the fundamental performance and security guarantees of onion-based anonymous routing in DTNs. Moetesum et al. in Ref. [21] present an adaptive and efficient buffer management scheme called size-aware drop that strives to improve buffer utilization and avoid unnecessary message drops.

Those protocols suffer from the problem of a continuous spread of messages even though they are already delivered to a destination. This drawback leads to bandwidth overhead and high resource usage on the entire network. Generally, existing routing protocols in DTN can be classified into some categories: flooding-based protocol, quota-based protocol, copy-based routing and so on. Some of them achieve high delivery rates, but expend excessive network resources, while some of them reduce the overhead, but they are not able to obtain comparable delivery rates. 

Our proposed protocol is a type of message ferrying routing protocol based on PROPHET. FIFO (First Input First Output) queuing is the nature of PROPHET. When a new message enters the full buffer of node, messages with the longest storage time will be dropped consistently. Messages may be lost due to network failures or forwarding to a few concentrated nodes. During the above situations, transmission may be delayed. Therefore, there is a variety of different routing protocols proposed to improve the performance of PROPHET. Similar to our study, Refs. [22,23,24,25,26] modified or appended some parameters to achieve their goals based on PROPHET. In message ferrying, the source node evaluates all contacting nodes with the predictability of successful delivery by using defined functions and transmits messages to the node with the highest delivery rate. In order to reduce messages dropping and transmission delay in DTN, we try to make use of predictability and buffering to improve routing performance in this study.

## 3. Methods

In DTN, the major task of routing is to decide the replicated nodes. Therefore, DTN routing methods are very important because they give influence to communication performance. After analyzing the drawbacks of PROPHET, we try to improve the routing performance by providing two scheduling mechanisms [27]. We propose an improved version of the PROPHET routing protocol based on scheduling, called Scheduling-PROPHET, which can perform better in buffering and transmission. The details will be described in the following sub-sections. In this section, we mainly describe our assumptions and the protocol in detail.

**Figure 1:**
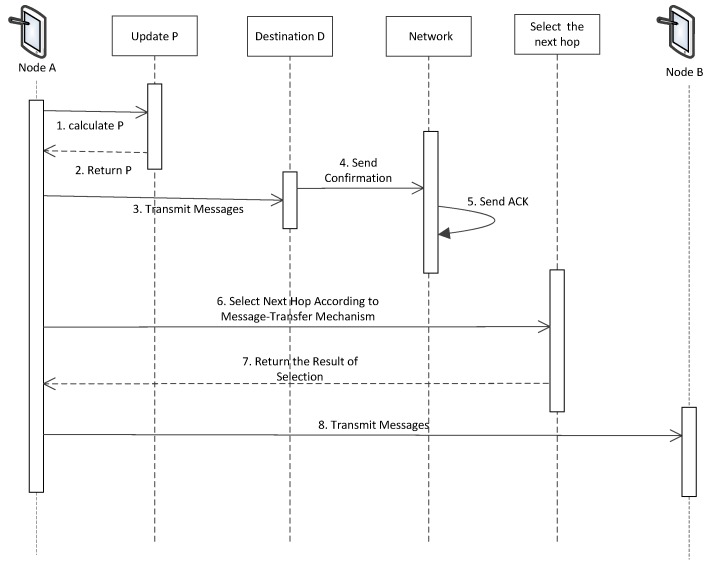
A sequence chart for the major procedure of Scheduling-Probabilistic Routing Protocol using History of Encounters and Transitivity (PROPHET).

In a realistic IoT application, DTNs are typically characterized by the unguaranteed connectivity and the low frequency of encounters between a pair of nodes. Scheduling-PROPHET uses several scheduling mechanisms to improve the protocol in transmitting and managing messages. Figure 1 briefly illustrates the procedure of the proposed routing protocol. 

### 3.1. Network Model

In this sub-section, we present a basic network model for formalizing the operations of Scheduling-PROPHET. 

*Nodes:* We assumed that each node has a unique nonzero identifier i, which is bound to a public identity. Furthermore, the buffer space for carrying messages originating from others is limited, but the buffer space for itself is unlimited. For IoT applications, this restriction on buffer space or storage is quite important. As long as one node encounters any other node, they can get a transfer opportunity. All node movement is random so that they cannot estimate when they will get the next opportunity. Utilizing delivery predictability is the main idea of our routing protocol. It will be calculated, if two nodes encounter each other in the communication range. In addition, each node keeps a table locally, which contains its identification, predictability, received messages, and traffic load, etc. Each node will update their traffic load every 30 s. Just as shown in Figure 1, when two nodes encounter each other, they will exchange the predictability P in their tables and then update the predictability in the network according to Equations (1) and (2). 

*Transfer:* The transfer opportunity is assumed to be limited in both duration and bandwidth, due to the node mobility. Nodes are willing to discover and transmit messages to each other in the communication range. However, the number of messages that can be transferred is restricted. In our protocol, the selection of transmission depends on the complete delivery predictability (S(i,i+1,…,D)), the hops (h(i,i+1,…,D)) and the traffic load ($tl_{i}$). As shown in Figure 1, messages will be transmitted to the destination nodes firstly without doubt. To other nodes (except the destination), we will compare the available paths and select a better one. Scheduling-PROPHET selects the path with the highest delivery predictability to transmit messages. If there are two or more paths ranked with the highest delivery predictability, Scheduling-PROPHET will choose the path with the minimum number of hops. In real-life applications, if there are still two or more paths ranked with the highest delivery predictability and minimum number of hops, Scheduling-PROPHET has to take the traffic load into consideration. We will compare the traffic load of the next hops, and preferentially choose the next hop with the minimum traffic load.

*Storage:* Each node is required to manage its own limited buffer space, by assigning priority to messages and deleting messages according to the protocol. In addition, each node will update its traffic load and keep this information in a table. Scheduling-PROPHET will choose the next hop with less traffic load. In this way, it will increase the delivery rate of the whole network, and messages will get more opportunity to be delivered. We assume that each node must receive messages in an application layer and remove it from the buffer. Each node destines to carry all messages and forward the copies of messages to other nodes until the message is notified of delivery by acknowledgment (ACK). 

We assume that messages will be deleted only in the following three cases, except for some unpredictable events like dropping during the transmission:

Case 1: After the messages have been transmitted to the destination and all senders get ACK form network, nodes are able to remove the message from their buffer spaces.

Case 2: Time is up for messages to stay in the buffer space. The TTL of messages is set to 300 s (about 5 h) in our simulation.

Case 3: When the buffer space is full, messages with the lowest priority will be deleted.

### 3.2. Protocol Definition

In order to overcome the drawbacks of network failures, by which a connected path from source to destination is never available, we proposed a new routing protocol called Scheduling-PROPHET. Calculating delivery predictability by the encountering frequency is the key point of the Scheduling-PROPHET routing protocol. According to the delivery predictability, we can deduce whether a message should be transmitted or removed from the ranked list of the nodes’ carried messages. In addition, Scheduling-PROPHET uses acknowledgments to notify all nodes that messages have been delivered to. In the remainder of this sub-section, we are going to explain the delivery predictability and transmitting management with scheduling in detail.

We use the incremental averaging method to make the change of delivery predictability smoother. The calculation model for delivery predictability is given as follows. We establish a probabilistic value called delivery predictability, noted as P(A,B)∈[0,1]. It refers to the probability that node A encounters node B in the network. To each node, it is required to preserve a table to store information about delivery predictability. When node A encounters node B, they will exchange their table information to determine whether to transfer messages or not. We set an initial value called $P_{init}\in (0,1]$, which is an initialization constant for each node.

When node A encounters node B, the delivery predictability is calculated by Equation (1), where β∈(0.5,1).

(1) $$P{\left(A,B\right)}_{new}=P{\left(A,B\right)}_{old}+[1-P{\left(A,B\right)}_{old}]*\beta$$

After that, the delivery predictabilities of all nodes will be recalculated according to Equation (2), where N denotes the set of all nodes.

(2) $$P(i,j)=\frac{P{\left(i,j\right)}_{old}}{2},\;(i,j\in N)$$

When node A encounters node B for the first time, the delivery predictability is calculated by Equation (3).

(3) $$P{\left(A,B\right)}_{new}=P_{init}$$

Therefore, P is directly related to the frequency by which they meet each other. According to Equation (2), if two nodes cannot encounter each other, the value of P will become much lower.

In the worst case, forwarding messages to intermediate nodes that rarely encounter the destination node will cause delivery failure. Therefore, we try to calculate the complete delivery predictability from the current node to the destination node. The complete delivery predictability is calculated by Equation (4), where S(i,i+1,…,D) denotes the total predictability from the current node i to the destination node D.

(4) $$S(i,i+1,\ldots ,D)=\prod_{x=i}^{D-1}P(x,x+1).$$

**Figure 2:**
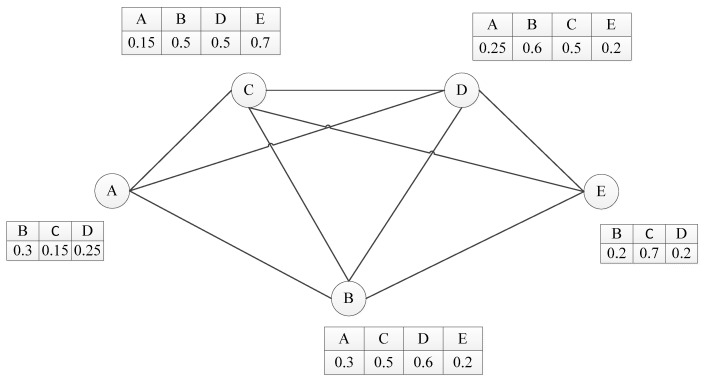
An example of a selecting transmission path.

From the source node A to the destination node B, we can get a series of values for S(i,i+1,…,D) by Equation (4), and then we can choose an appropriate path with the highest value for S(i,i+1,…,D). In this way, we are able to choose the most efficient transmission path, to improve the transmission efficiency and reduce the message drop rate. Figure 2 shows an example illustrating the working process of our routing protocol. In this example, each node holds the delivery predictabilities. There are eight connected paths between node A (source node) and node E (destination node), and the list of paths is as follows:

(1) S(ABE)=P(A,B)∗P(B,E)=0.3∗0.2=0.06
(2) S(ACE)=P(A,C)∗P(C,E)=0.15∗0.7=0.105
(3) S(ADE)=P(A,D)∗P(D,E)=0.25∗0.2=0.05
(4) S(ABCE)=P(A,B)∗P(B,C)∗P(C,E)=0.3∗0.5∗0.7=0.105
(5) S(ABDE)=P(A,B)∗P(B,D)∗P(D,E)=0.3∗0.6∗0.2=0.036
(6) S(ACDE)=P(A,C)∗P(C,D)∗P(D,E)=0.15∗0.5∗0.2=0.015
(7) S(ADBE)=P(A,D)∗P(D,B)∗P(B,E)=0.25∗0.6∗0.2=0.03
(8) S(ABCDE)=P(A,B)∗P(B,C)∗P(C,D)∗P(D,E)=0.3∗0.5∗0.5∗0.2=0.015

We can see that the path ACE is an optimal one because of its high predictability (S(ACE)=0.105). Thus, the source node A will transfer messages to node C according to the protocol. In fact, there are two paths that have high predictability (S(ACE)=S(ABDE)=0.105), and then we will compare the hop count between the paths (the detail is given in Section 3.3). In this example, the hop count h(ACE) and h(ABDE) are 2 and 3, respectively. Then, the message will be transmitted from node A to node C by the path with fewer hops.

### 3.3. Transmitting Messages

To solve the problem on dropping messages and increase the delivery rate on significant messages, we design two scheduling mechanisms: *Message Management Mechanism* and *Message-Transfer Mechanism*.

*Message Management Mechanism*: In order to make the bandwidth more effective and save buffer space, we set some criteria to coordinate the priority of in-transit messages. The so-called Message Management refers to managing the messages stored in buffers. The core of the management involves dropping messages and assigning priority. All nodes in the network are notified by ACK and then remove the delivered messages from their buffers. If the messages have lower delivery predictability, we assign lower priority to them in order to restrain their transmission. If the buffer space is full, messages with the lowest priority will be removed to make more room for forthcoming messages. In this way, the routing protocol avoids generating copies for the messages with low priority and improves the utilization of network resources.

*Message-Transfer Mechanism*: In Scheduling-PROPHET, when node I encounters node J, I will transmit messages to J. The Messages-Transfer Mechanism is illustrated as follows. Firstly, I transmits messages to J which is the destination of those messages. Secondly, I exchanges the table that holds the delivery predictabilities of all nodes with J, and decides the transmission path. The determinate factors for the path are complete predictability (S(i,i+1,…,D)), the amount of hops of path (h(i,i+1,…,D)) and traffic load ($tl_{i}$). Here choosing a transmission path with the largest S(i,i+1,…,D) means choosing the path with the highest delivery predictability. When two or more paths have the same value of delivery predictability, we will check the hop count of path (h(i,i+1,…,D)). A transmission path with minimum hops means the shortest path. However, there may still exist two or more paths with the same hop count, and then we will compare the traffic load of all possible next hops ($tl_{i}$), and choose the node with the least traffic load as the next hop of the transmission path.

The Algorithm 1 for Scheduling-PROPHET routing protocol to explain the procedure is given as follows. Nodes will exchange their lists, and transmit messages following the Scheduling-PROPHET routing protocol.
**Algorithm 1.** Scheduling-PROPHET1: for each node A:2:  when node A encounters node B, they exchange their tables;3:  $P{\left(A,B\right)}_{new}=P{\left(A,B\right)}_{old}+[1-P{\left(A,B\right)}_{old}]*\beta$4: end for;5: for each node pair *i* and *j*:6:   $P(i,j)=\frac{P{\left(i,j\right)}_{old}}{2}$, (i,j∈N)7: end for;8: for each message M of A:9:  if(D==B){10:   A forwards M to B;11:   D notifies that M has arrived and it can be deleted from all node buffers;12:  }13:  else{14:   A exchanges table with B and gains the set of connected paths (denoted as L);15:    if(L≠NULL){16:     Arrange the paths in L from high to low according to the value of S;17:     Get the top three paths $L_{1},L_{2},L_{3}$ from L18:      if($S(L_{1})=S(L_{2})∥S(L_{1})=S(L_{2})=S(L_{3})$){19:       A calculates the hops of $L_{1}$, $L_{2}$, $L_{3}$ respectively;20:       if($h(L_{1})=h(L_{2})∥h(L_{1})=h(L_{2})=h(L_{3})$){21:        A compares the traffic load of next hop on $L_{1}$, $L_{2}$, $L_{3}$ respectively;22:        A forwards M to the next hop with the least traffic load;23:       }24:      }25:      else {26:      A forwards M according to $L_{1}$;27:     }28:   }29:    else {30:     do nothing;31:    }32:  }33: end for;

The notations used in the above algorithm are listed as follows:

- A: a node carrying messages (M);
- B: a node encountered by A;
- M: the message node A intends to send;
- D: the destination of M;
- K: any other node that is not encountered by A;
- N: the set of all nodes;
- $L_{i}$: the connected path from source to destination;
- $S(L_{i})$: the complete predictability of $L_{i}$.

We explain the process of the algorithm further as follows:

(1) The value of P(A,B) is calculated by Equation (1), and then all P values are re-normalized by Equation (2).
(2) Messages are transmitted to the encountered node based on its priority. Specifically, if node A discovers the destination (node D) of the message (M), A will transmit M to D firstly. Otherwise, transmission is based on a ranked list of messages stored in nodes.
(3) The message receiver (node D) confirms that it has already received the correct message, and notifies ACK to all senders.
(4) Messages that have not been transferred in the buffer space will be transmitted one by one according to the priority. Scheduling-PROPHET calculates the possible paths (L) from the current sender to the destination according to the complete delivery predictability. In the protocol, we denote the maximum amount of paths to compare as Δλ. Meanwhile, each possible path is normalized by Equation (4). For all paths, we rank them from high predictability to low based on the value of S(i,i+1,…,D). Scheduling-PROPHET selects the path with the highest predictability for transmission.
(5) We found that there will be more than one path with the highest predictability in real-life applications, but Scheduling-PROPHET forbids choosing two or more paths concurrently. Therefore, we attempt to take an additional factor, the hop count of a path (h(i,i+1,…,D)). To the paths with the same predictability, the hop count of a path is calculated by Scheduling-PROPHET, and those paths are arranged in order according to their hop counts. Scheduling-PROPHET will choose the path with the minimum hops to transmit messages. In our protocol, we set the maximum amount of paths for comparison to 3.
(6) Finally, in real applications, traffic load is a significant factor that has an impact on transmission efficiency. Thus, in our protocol, we take traffic load ($tl_{i}$) into consideration. For the paths with the same hop count, the traffic load kept by the nodes will be compared. Scheduling-PROPHET will choose the next hop with the least traffic load to transmit messages.

In summary, Scheduling-PROPHET keeps choosing paths with the highest predictability for successful delivery, the minimum hop count and the least traffic load. In this way, we are able to use the most efficient transmission path to improve transmission efficiency and reduce message drop rate and delay.

## 4. Results and Discussion

We have a series of results after the simulation on an Opportunistic Network Environment (ONE) simulator. We can see the performance of the proposed protocol compared with the other two protocols according to the results. We will make a discussion on the results in detail in the following sub-sections.

### 4.1. Simulation Setting

We simulate our proposed routing protocol in ONE [28] simulator. The ONE simulator is implemented by Java and available as open source. Compared with other DTN simulators, the major advantage of the ONE simulator is that we can extend it to evaluate new protocols and algorithms including the proposed protocol in this study. As an open source project, it allows users to create scenarios based upon different synthetic movement models and real-world traces and offers a framework for implementing routing protocols. It supports simulating the mobility pattern of the nodes and the message exchange between them. Moreover, ONE simulator provides a variety of report types and analyzing modules. 

In this study, we choose an appropriate scenario to simulate our routing scheme for message transmitting. In order to evaluate the performance of Scheduling-PROPHET, we compare it with two typical protocols, Epidemic and PROPHET. In our simulation, we mainly focus on evaluating the performance with four metrics: Delivery Ratio, Delivery Delay, Overhead, Hop Count and Number of Dropped Packets [29,30]. Our implementations run with the following settings (see Table 1). The mobile nodes are divided into six groups and move in a 4500 × 3400 m^2^ area of Helsinki city, which is a default scenario in ONE. Group1 and Group3 move with a speed uniformly about 0.5–1.5 m/s, as the pedestrians following the “ShortestPathMapBasedMovement” movement model. Group2 moves with a speed uniformly about 2.7–13.9 m/s, as an automobile driving on road. Group4, Group5 and Group6 move with a speed uniformly about 7–10 m/s, as trams driving on the given road—“MapRouteMovement”. In our implementation, the speed for transmitting messages is 250 k/s with Bluetooth. We set Δλ≤10 for comparing the possible paths. Then, we evaluate the protocols by the following three specific scenarios:

- Evaluating the performance on delivery rate, overhead and the number of dropped packets, when the transmission radius varies from 4 m to 10 m according to the Bluetooth protocol.
- Evaluating the performance on delivery rate, delivery delay, overhead, hops and the number of dropped packets, when the node density varies from 12 to 645 in the network.
- Evaluating the performance on delivery rate, delivery delay, overhead, hops and the number of dropped packets, when the buffer size varies from 10 MB to 90 MB on each node.

### 4.2. Transmission Range

In our simulation, nodes transmit messages by using Bluetooth. In an environment with obstacles, the maximum transmission distance of Bluetooth is about 10 m. If we denote the transmission distance as r meters, the transmission range is a circle with a radius of r meters. Thus, we ran the simulation program to evaluate how our routing protocol performs under different transmission ranges. In our evaluation, we change the transmission radius varying from 4 m to 10 m, and we ran 20 times with each transmission radius for each protocol. Meanwhile, there are 126 nodes in the whole network, and we assign a buffer space of 5 M to pedestrians and automobiles, 50 M to trams.

**Figure 3:**
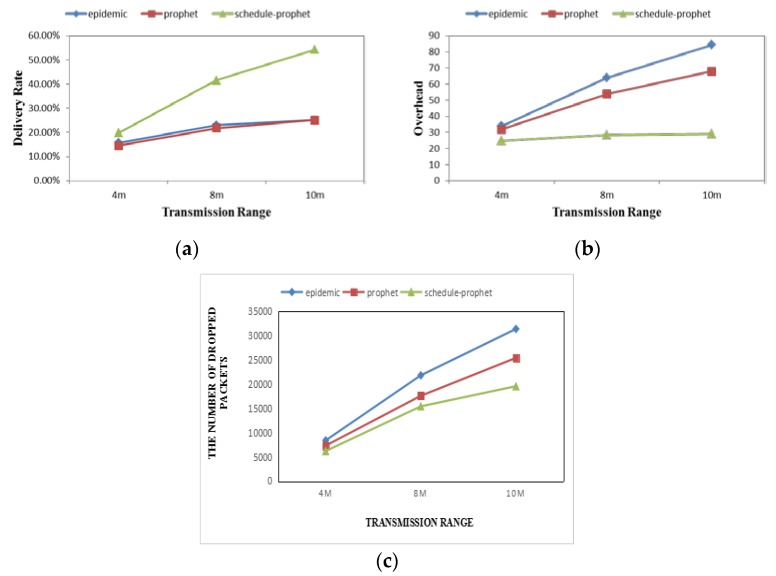
Performance comparison under different transmission ranges with two metrics: (**a**) Delivery Rate; (**b**) Overhead; (**c**) Dropped Packets.

Figure 3 shows the performance of three protocols under different transmission ranges. Figure 3a–c depicts that, even in a relatively short transmission range, Scheduling-PROPHET still gains better performance with a higher delivery rate, lower overhead and lower packet drop rate than others. This implies that our protocol can keep better transmission efficiency than others, with less cost.

### 4.3. Nodes Distribution

We model 10–700 mobile nodes to evaluate adaptability in different densities. The node distribution, buffer space and transmission range are shown in Table 2. In this part, we ran 15 times with each simulation setting for each protocol.

**Figure 4:**
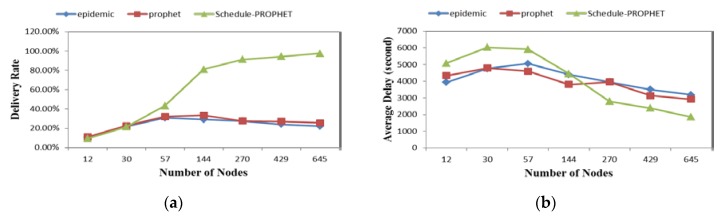

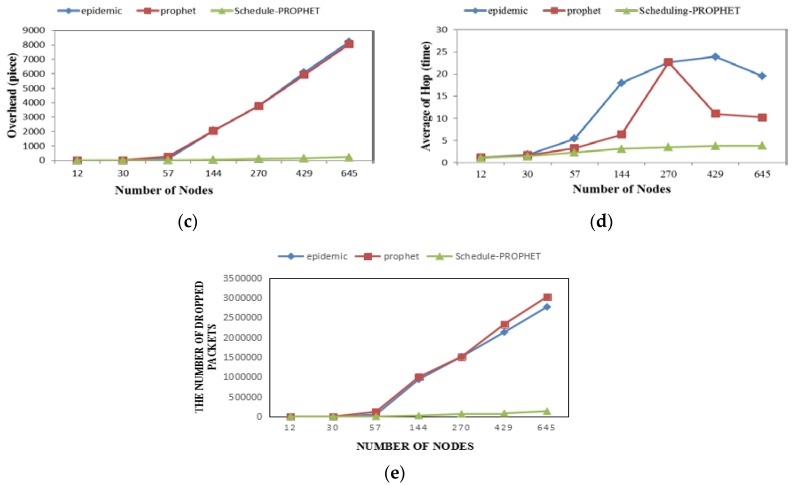
Performance comparison in different nodes distribution with four metrics: (**a**) Delivery Rate; (**b**) Delivery Delay; (**c**) Overhead; (**d**) Hop Counts; (**e**) Dropped Packets.

Figure 4 summarizes some key metrics of the simulation results with different density. The results of the delivery rate are shown in Figure 4a. Delivery rates of these three routing protocols are almost the same in low density. With the nodes increasing, our routing protocol gets higher delivery rates than others. Delivery delay means the time for messages to be transformed from source to destination. In our simulations, we take the average delivery delay as a metric of comparison to evaluate performance. In Figure 4b, we can find that Scheduling-PROPHET performs much better than others on average delivery delay at high density. But two traditional routing protocols show more advantages at low density. They use simplex and direct mechanisms to transfer messages, as described in Refs. [5,7]. That is why they cannot perform well at low density. Overhead refers to how many messages are transmitted successfully in a time unit. As we describe in Section 3.3, we proposed two improved mechanisms to make our routing protocol more efficient. It is easy to see that the proposed routing protocol gets the best performance on overhead and packet drop rate, as shown in Figure 4c,e. It benefits from keeping high delivery predictability. Moreover, Scheduling-PROPHET takes the hop count into consideration, and chooses the shortest path to transmit messages. The result in Figure 4d is validating the effectiveness as Figure 4c.

### 4.4 Buffer Size

In order to manifest how protocols react to the changes in different buffer size, we change the buffer space in the network from 10 M to 90 M as shown in Table 3, and we ran it 15 times with each simulation setting for each protocol.

**Figure 5:**
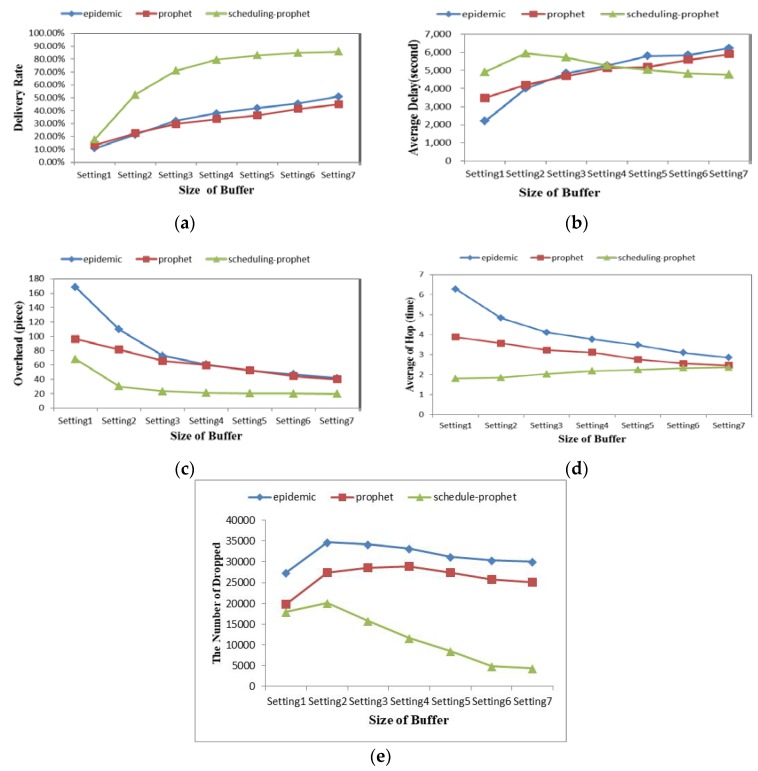
Comparison in different buffer size with four metrics: (**a**) Delivery Rate; (**b**) Delivery Delay; (**c**) Overhead; (**d**) Hop Counts; (**e**) Dropped Packets.

Figure 5 shows the performance of the three protocols with different buffer sizes. Figure 5a,c depicts that the performance of the three protocols are improved obviously when the size of the node buffer increases. In addition, Scheduling-PROPHET also performs better in this simulation. In Figure 5b, the delivery delay of the protocols except Scheduling-PROPHET increases when the size of the node buffer increases. We observe in Figure 5e that Scheduling-PROPHET has a much lower packet drop rate than others. The number of dropped packets in Scheduling-PROPHET decreases quickly with increasing buffer space. This is probably due to the fact that too many messages are blocked in buffer, while we use scheduling to make transmission more efficient in Scheduling-PROPHET. The evaluation on hop count is given in Figure 5d. With the increasing of the buffer size, the hop count of Scheduling-PROPHET, Epidemic and PROPHET almost converge towards the same level.

### 4.5. Message Generation Interval

In this section, we further evaluate the delivery rate and overhead with different message generation intervals. We also compare our protocols with two protocols: Hybrid Type routing [23] and PRoPHET+ routing [25]. The simulation parameters we used are shown in Table 4.

**Figure 6:**
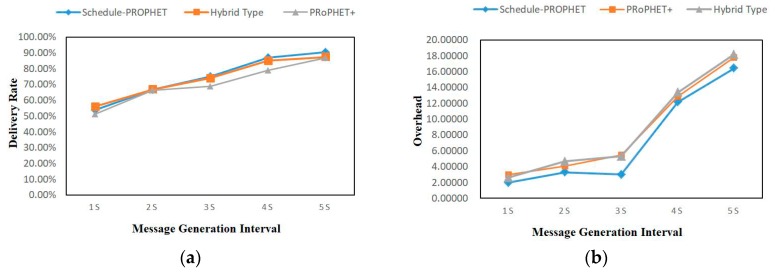
Comparison in different intervals of message generation with the metrics: (**a**) Delivery Rate; (**b**) Overhead.

As we can observe in Figure 6a, the proposed protocol performs a little bit better than both Hybrid Type and PRoPHET+. In the proposed protocol, since delivery predictability is used to choose intermediate nodes, it can avoid over-replication, and overflow in storage. That is why the delivery rate of Scheduling-PROPHET can increase, even though the network load is high. From Figure 6b, we can see that the proposed protocol has got a lower overhead, because it assigns priority for messages by using the message management mechanism (described in Section 3.3).

### 4.6. Simulation Summary

From the simulation results above, we can conclude that Scheduling-PROPHET achieves a better performance in several scenarios. First, our protocol gets better performance in different transmission ranges, compared with other protocols. Even in a relatively short transmission range, the protocol still gains better performance than others. The protocol also performs much better than others in the case of high node density in a network. Moreover, our protocol gets better performance than others when the buffer size increases. It means that we could consider increasing the buffer size of nodes if we want to make full use of the protocol in data transmission.

A big challenge for IoT applications is to prolong network lifetime and maintain connectivity with resource limitation in order to deliver data to its destination. DTNs seem to be a suitable solution to handle intermittent connectivity, overcome resource constraints and network disconnection for IoT applications. Therefore, the proposed protocol can be used to achieve efficient data transmission in IoT scenarios, e.g., a mobile sensor network where sensor nodes are not powerful enough to send data to a collecting station all the time or scheduled to wake/sleep periodically to save their battery consumption. Scheduling-PROPHET can be implemented to satisfy the data transmission requirements of IoT applications. Compared with existing protocols, we can see that the proposed protocol can be used well in IoT applications where the underlying network is characterized by intermittent connectivity, dynamic mobility and resource constraints. All we need to do is extend the existing PROPHET protocol with our mechanisms. However, according to the simulation results, it would be better to have a large buffer for our protocol in order to get a better performance in real-life IoT applications. Moreover, it will be better if the data transmission is based on Bluetooth.

## 5. Conclusions

In this study, we introduce the Scheduling-PROPHET, which utilizes two scheduling mechanisms that improved the performance of the routing protocol in DTN for IoT applications. The major goal of Scheduling-PROPHET is to achieve a high delivery rate and reduce delivery delay while minimizing resource consumption in transmission. The other main idea of the protocol is to manage the messages in a buffer. After introducing the details about the routing protocol, we further illustrate the simulations by using the ONE simulator to evaluate the performance with five metrics (Delivery Ratio, Delivery Delay, Overhead, Hop Count and Number of Dropped Packets). Through the simulation results, we can see that the proposed protocol performs much better than others.

## Figures and Tables

**Table 1:** Common parameters for the simulation.

	Parameter	Value		
Scene	Simulated City	Helsinki city		
	Simulation Time	24 h		
	Simulation Area	4500 m × 3400 m		
Messages	Messages TTL	5 h		
	Messages Size	500 KB–1 MB		
	Generating Interval	25–35 s		
Nodes	Grouping	Pedestrians	Automobiles	Trams
	Movement Model	ShortestPath MapBased Movement	ShortestPath MapBased Movement	MapRoute Movement
	Transmission Interface	Bluetooth		
	Moving Speed	0.5–1.5 m/s	2.7–13.9 m/s	7–10 m/s
Bluetooth	Transmit Speed	250 KB/s		

**Table 2:** Simulation parameters for node distribution.

Parameter			Value		
Messages	Generating Interval		5 s		
Nodes	Grouping		Pedestrains	Automobiles	Trams
	Buffer Space		5 M	5 M	50 M
	Nodes Distribution	Setting1	6	3	3
		Setting2	16	8	6
		Setting3	30	15	12
		Setting4	80	40	24
		Setting5	160	80	30
		Setting6	260	130	39
		Setting7	400	200	45
Bluetooth	Transmission Range		10 m	10 m	10 m

**Table 3:** Simulation parameters for buffer space.

Parameter			Value		
Messages	Generating Interval		5 s		
Nodes	Grouping		Pedestrians	Automobiles	Trams
	Buffer Space	Setting1	2 M	2 M	10 M
		Setting2	5 M	5 M	25 M
		Setting3	8 M	8 M	40 M
		Setting4	10 M	10 M	50 M
		Setting5	12 M	12 M	60 M
		Setting6	15 M	15 M	75 M
		Setting7	18 M	18 M	90 M
	Nodes Distribution		80	40	24
Bluetooth	Transmission Range		10 m	10 m	10 m

**Table 4:** Simulation parameters for message generation intervals.

Parameter		Value		
Nodes	Grouping	Pedestrians	Automobiles	Trams
	Buffer Space	5 M	5 M	50 M
	Nodes Distribution	80	40	24
Bluetooth	Transmission Range	10 m	10 m	10 m
